# High-density lipoprotein-cholesterol and antipsychotic medication in overweight inpatients with schizophrenia: post-hoc analysis of a Japanese nationwide survey

**DOI:** 10.1186/s12888-018-1764-1

**Published:** 2018-06-08

**Authors:** Shin Ono, Takuro Sugai, Yutaro Suzuki, Manabu Yamazaki, Kazutaka Shimoda, Takao Mori, Yuji Ozeki, Hiroshi Matsuda, Norio Sugawara, Norio Yasui-Furukori, Kurefu Okamoto, Toyoaki Sagae, Toshiyuki Someya

**Affiliations:** 1 Department of Community Psychiatric Medicine, Niigata University Graduate School of Medical and Dental Sciences, Niigata, Japan; 2 Japanese Society of Clinical Neuropsychopharmacology, Tokyo, Japan; 3 Department of Psychiatry, Niigata University Graduate School of Medical and Dental Sciences, Niigata, Japan; 4 Japan Psychiatric Hospital Association, Tokyo, Japan; 5 Department of Psychiatry, Dokkyo Medical University School of Medicine, Mibu, Japan; 6 Department of Neuropsychiatry, Hirosaki University School of Medicine, Hirosaki, Japan; 7 Department of Health and Nutrition, Yamagata Prefectural Yonezawa University of Nutrition Sciences Faculty of Health and Nutrition, Yonezawa, Japan

**Keywords:** Schizophrenia, Antipsychotics, High-density lipoprotein-cholesterol, Metabolic syndrome

## Abstract

**Background:**

Patients with schizophrenia have an increased prevalence of metabolic disturbances compared with the general population. However, the mechanisms underlying the metabolic side effects of antipsychotics are unknown. The aim of the present study was to compare the levels of high-density lipoprotein (HDL)-cholesterol in Japanese schizophrenia patients medicated with olanzapine, risperidone, or aripiprazole monotherapy.

**Methods:**

This study was a post-hoc analysis of a nationwide survey, which included 433 Japanese outpatients with schizophrenia and 674 inpatients. A brief questionnaire was compiled that covered demographic data, systolic blood pressure, diastolic blood pressure, and HDL-cholesterol after reviewing the relevant literature and guidelines. To compare demographic and clinical characteristics, analysis of variance was performed for continuous variables and the chi-square test was performed for categorical variables. For comparisons of HDL-cholesterol levels among the three antipsychotic groups, analysis of covariance was carried out with age, diastolic blood pressure, chlorpromazine-equivalent dosage, and waist circumference as confounding variables after stratification by body mass index (BMI) for each outpatient group and inpatient group.

**Results:**

The mean age was 57.9 ± 14.0 years and the mean BMI was 23.4 ± 4.5 kg/m^2^. HDL-cholesterol levels when stratified by BMI differed significantly (*p* = 0.019) between the three antipsychotic groups after age, diastolic blood pressure, chlorpromazine-equivalent dosage, and waist circumference in inpatients. A significant difference in HDL-cholesterol levels was only found in the overweight inpatient group, and no significant differences in HDL-cholesterol levels were found among the three antipsychotics for outpatients of all BMI stratifications or inpatients that were underweight or of normal weight. For post-hoc analysis of HDL-cholesterol levels in overweight inpatients, HDL-cholesterol was significantly lower in the olanzapine group than in the aripiprazole group (*p* = 0.023).

**Conclusions:**

This study reveals a difference in HDL-cholesterol levels in overweight Japanese inpatients with schizophrenia resulting from the use of different antipsychotics. In the post-hoc analysis of HDL-cholesterol levels in overweight inpatients, HDL-cholesterol was significantly lower in the olanzapine group than in the aripiprazole group. Further studies incorporating more detailed evaluations, including diet and physical activity, are needed to clarify the differences in HDL-cholesterol according to antipsychotic use.

## Background

Patients with schizophrenia suffer from increased morbidity and mortality compared with the general population and have a life expectancy that is approximately 20% shorter [1]. Patients with schizophrenia also have an increased prevalence of cardiometabolic risk factors such as obesity, diabetes type 2, hypertension, hyperglycemia, and dyslipidemia compared with the normal population [2–4]. Low high-density lipoprotein (HDL)-cholesterol level, a cardiometabolic risk factor, is strongly and inversely associated with the risk of coronary heart disease [5]. Some studies attributed metabolic disturbance in schizophrenia to poor patient lifestyle choices such as sedentary lifestyle, smoking, poor diet, prolonged stress, as well as use of antipsychotic drugs, and genetic susceptibility [6].

Metabolic disorders associated with atypical antipsychotic drugs include abnormalities of glucose metabolism such as insulin resistance, hyperglycemia, dyslipidemia and weight gain [7–11]. In a meta-analysis study of second-generation antipsychotics, olanzapine increased cholesterol levels to a level higher than that induced by aripiprazole, risperidone, or ziprasidone [12]. However, the underlying causal mechanisms of metabolic side effects with antipsychotics are unknown. Apart from the effects of antipsychotics related to lipid abnormalities, patients with schizophrenia who are antipsychotic drug-naïve, or drug-free, also have low levels of HDL-cholesterol and are at high risk for metabolic syndrome [13].

Many confounding factors affect HDL-cholesterol levels. It is well known that HDL-cholesterol levels are decreased in overweight subjects [14, 15], and there is a negative association with body mass index (BMI) and HDL-cholesterol [16]. Additionally, smoking decreased HDL-cholesterol [17] while exercise increased HDL-cholesterol [18, 19]. As these various factors (e.g. diet, exercise, smoking, obesity, antipsychotics, and schizophrenia) influence HDL-cholesterol, it seems prudent to investigate HDL-cholesterol in schizophrenia in relation to BMI.

The prevalence of metabolic syndrome in schizophrenia patients treated with antipsychotics is higher than in the general population. In a previous nationwide survey on the prevalence of metabolic syndrome in the Japanese population, the prevalence in outpatients was approximately 3-fold higher than that in inpatients [20]. Japan has more psychiatric hospitals than other Organization for Economic Cooperation and Development (OECD) countries [21]. The average length of stay in psychiatric beds was 301.0 days according to surveys by the Ministry of Health, Labour and Welfare in Japan [22], and the length of stay in psychiatric beds in Japan was considered longer than that in other countries. The system for psychiatric treatment in Japan is different from other countries in terms of longer stay in psychiatric beds and higher number of psychiatric beds. Of note, there is a relatively uniform diet or exercise regime in a hospitalized environment compared with the outpatient environment in Japan. Drug adherence is also maintained in hospitals. Therefore, differences in medical care environments, such as hospitalization and outpatient treatment, should be taken into account when evaluating HDL-cholesterol in schizophrenic patients, especially in Japan.

The aim of the present study was to compare HDL-cholesterol levels between Japanese schizophrenia patients medicated with olanzapine, risperidone, or aripiprazole monotherapy as a post-hoc analysis of a previous nationwide survey.

## Methods

### Subjects

This study was a post-hoc analysis of a previous survey [20]. Of the 23,116 subjects in the original survey, only those receiving monotherapy with olanzapine, risperidone, or aripiprazole were included in the present study. The original sample included 7655 outpatients and 15,461 inpatients diagnosed with schizophrenia who had been taking the same antipsychotics. All of the subjects were diagnosed with schizophrenia based on International Statistical Classification of Diseases and Related Health Problems version 10 (ICD-10) or the Diagnostic and Statistical Manual of Mental Disorders, fourth edition, text revision (DSM-IV-TR) [23, 24]. The study protocol and informed consent procedure were approved by the Ethics Committee of the Japan Psychiatric Hospitals Association. The investigation was carried out in accordance with the Declaration of Helsinki. Each participant gave written informed consent prior to all study procedures.

The number of Japanese patients with schizophrenia was 795,000, and the majority of these patients were treated in hospitals in the Japan Psychiatric Hospitals Association in an investigation conducted by the Ministry of Health, Labour and Welfare in 2008. For the aim of assisting patients with schizophrenia, a joint project with the cooperation of the Japanese Society of Clinical Neuropsychopharmacology and the Japan Psychiatric Hospitals Association was started in Japan in 2012.

A questionnaire survey was conducted between January 2012 and July 2014. Responses were obtained from 7655 outpatients and 15,461 inpatients in 520 facilities for outpatients and 247 facilities for inpatients belonging to the Japan Psychiatric Hospitals Association. We excluded patients aged under 20 years; patients whose sex, BMI, and HDL-cholesterol data were not recorded; and patients medicated with polypharmacy of antipsychotics or monotherapy with drugs other than olanzapine, risperidone, or aripiprazole. The final study population comprised 1107 individuals.

### Assessments

A brief questionnaire was compiled to cover demographic data (age and sex), systolic blood pressure, diastolic blood pressure, and HDL-cholesterol after reviewing the relevant literature and guidelines. Body weight, height, and waist circumference were measured, and BMI was calculated by weight (kg)/height squared (m^2^). Blood pressure was measured twice with patients in a seated position after at least 5 min of rest, using a standard mercury sphygmomanometer. HDL-cholesterol was measured by standard analytical techniques.

### Statistical analyses

Demographic and clinical variables are presented as the mean ± standard deviation (SD) or proportion. To compare demographic and clinical characteristics between the three antipsychotic groups, analysis of variance was performed for continuous variables and the chi-square test was performed for categorical variables. The threshold for significance was set at *p* < 0.05. SPSS Statistics 24 for Mac OS (IBM Japan, Tokyo, Japan) was used for statistical analyses. For comparisons of HDL-cholesterol levels among the three antipsychotic groups, analysis of covariance was carried out with age, diastolic blood pressure, chlorpromazine-equivalent dosage, and waist circumference as confounding variables after stratification by BMI (BMI < 18.5, underweight; 18.5 ≤ BMI < 25, normal weight; BMI ≥ 25, overweight) for each outpatient group and inpatient group. Post-hoc analyses were performed using the Bonferroni method.

## Results

There were 433 outpatients and 674 inpatients with schizophrenia in this study. All were Japanese with a mean age of 57.9 ± 14.0 years and a mean BMI of 23.4 ± 4.5 kg/m^2^. Table 1 summarizes the demographic and clinical characteristics of the entire study population (*n* = 1107). No significant intergroup differences were observed for sex, BMI, waist circumference, systolic blood pressure, or HDL-cholesterol. Significant differences were observed for age (*p* < 0.001), diastolic blood pressure (*p* = 0.025), and chlorpromazine-equivalent dosage of current atypical antipsychotics (*p* < 0.001). The HDL-cholesterol levels for overweight inpatients differed significantly among the three antipsychotic groups (*p* = 0.019) after adjustment for age, diastolic blood pressure, chlorpromazine-equivalent dosage, and waist circumference. For post-hoc analysis of HDL-cholesterol levels in overweight inpatients among the three antipsychotic groups, HDL-cholesterol was significantly lower in the olanzapine group than in the aripiprazole group (*p* = 0.023), and HDL-cholesterol levels were similar between the olanzapine and risperidone groups (*p* = 0.160). HDL-cholesterol levels in underweight and normal weight patients did not differ significantly (Table 2). No significant differences in HDL-cholesterol levels were found among the three antipsychotics for outpatients of all BMI stratifications (Table 3).

**Table 1:** Comparisons of demographic and clinical characteristics of patients in the three antipsychotic groups

		Olanzapine (*N* = 356)	Risperidone (*N* = 529)	Aripiprazole (*N* = 222)	*p*-value
Age	years	56.4 ± 14.0	60.2 ± 13.2	54.8 ± 15.2	< 0.001
Females (males)		158 (198)	243 (286)	115 (107)	0.201
Outpatients (inpatients)		138 (218)	194 (335)	101 (121)	0.077
BMI	kg/m^2^	23.2 ± 4.1	23.5 ± 4.5	23.5 ± 5.1	0.516
Waist circumference	cm	84.0 ± 11.5	84.8 ± 11.7	83.4 ± 14.1	0.297
Systolic blood pressure	mmHg	124.5 ± 18.9	122.7 ± 17.3	123.9 ± 19.1	0.355
Diastolic blood pressure	mmHg	77.2 ± 12.3	75.0 ± 12.5	74.9 ± 13.0	0.025
HDL-cholesterol	mg/dL	54.3 ± 18.6	55.4 ± 17.9	57.5 ± 17.9	0.126
CP-equivalent dosage of current AAP	mg	516.1 ± 261.7	420.8 ± 273.4	402.1 ± 231.7	< 0.001
Concomitant drugs					
Antidepressant		1	0	0	
Mood stabilizer		6	11	3	
Benzodiazepines		7	13	2	
Anticholinergic drug		0	9	0	

Data are shown as the mean ± SD or number of subjects

Age, BMI, waist circumference, systolic blood pressure, diastolic blood pressure, HDL-cholesterol, and CP-equivalent dosage of current AAP were compared among the three antipsychotics groups. The chi-square test was performed to analyze sex and outpatients (inpatients)

*BMI* body mass index, *HDL* high-density lipoprotein, *CP* chlorpromazine, *AAP* atypical antipsychotic

**Table 2:** Comparisons of HDL-cholesterol levels in the three antipsychotic groups with stratification by BMI for inpatients

			Olanzapine	Risperidone	Aripiprazole	*p*-value	Post-hoc (*p*-value)
HDL-cholesterol	BMI < 18.5	mg/dL	62.0 ± 20.5 (34)	58.6 ± 15.8 (55)	62.6 ± 21.6 (28)	0.490	
	18.5 ≤ BMI < 25	mg/dL	54.1 ± 21.5 (137)	55.0 ± 16.2 (196)	58.2 ± 15.8 (68)	0.282	
	BMI ≥ 25	mg/dL	43.8 ± 12.3 (47)	49.7 ± 15.0 (84)	54.7 ± 16.8 (25)	0.019	Olanzapine < Aripiprazole (0.023)

Data are shown as the mean ± SD (number of subjects)

Analyses of covariance among the three antipsychotics were performed for HDL-cholesterol with age, diastolic blood pressure, chlorpromazine-equivalent dosage and waist circumference as confounding variables

Post-hoc analyses were performed by the Bonferroni method

*HDL* high-density lipoprotein, *BMI* body mass index

**Table 3:** Comparisons of HDL-cholesterol levels in the three antipsychotic groups with stratification by BMI for outpatients

			Olanzapine	Risperidone	Aripiprazole	*p*-value
HDL-cholesterol	BMI < 18.5	mg/dL	72.1 ± 18.7 (8)	81.3 ± 30.4 (4)	62.5 ± 3.4 (4)	0.959
	18.5 ≤ BMI < 25	mg/dL	57.6 ± 13.4 (69)	61.4 ± 23.0 (96)	60.7 ± 17.7 (45)	0.442
	BMI ≥ 25	mg/dL	53.0 ± 15.6 (61)	52.7 ± 15.8 (94)	51.9 ± 18.4 (52)	0.819

Data are shown as the mean ± SD (number of subjects)

Analyses of covariance among the three antipsychotics were performed for HDL-cholesterol with age, diastolic blood pressure, chlorpromazine-equivalent dosage and waist circumference as confounding variables

Post-hoc analyses were performed by the Bonferroni method

*HDL* high-density lipoprotein, *BMI* body mass index

## Discussion

This study reveals that overweight inpatients with schizophrenia treated with antipsychotics had significant differences in HDL-cholesterol after adjustment for age, diastolic blood pressure, chlorpromazine-equivalent dosage, and waist circumference. However, there was no difference in HDL-cholesterol among the antipsychotic treatments in the overall patient group, outpatients of all BMI stratifications or inpatients of normal weight and underweight. We believe this survey is the first large-scale questionnaire survey in Japan to show a difference in HDL-cholesterol levels between antipsychotic drugs by BMI stratification.

Regarding the effects of antipsychotics on cholesterol, antipsychotic treatments have been reported to be associated with increased total and low-density lipoprotein (LDL)-cholesterol as well as decreased HDL-cholesterol [10, 25]. In a previous 52-week comparison study of olanzapine and aripiprazole, olanzapine was associated with significantly greater weight gain than aripiprazole throughout the study [26]. The change in HDL-cholesterol from baseline was significantly lower in the olanzapine group than in the aripiprazole group at the 52-week end-point where weight gain related to the two drugs was highest [26]. Although this prospective study by Chrzanowski and colleagues [26] was methodologically different to our cross-sectional study, our results also show that HDL-cholesterol is lower in overweight inpatients in the olanzapine group than those in the aripiprazole group. A 1-year observation study on first-episode drug-naïve patients revealed significant differences in HDL-cholesterol decreases between groups of patients who gained > 20% or < 20% of their baseline BMI at 1 year, and no significant difference in HDL-cholesterol between olanzapine- and risperidone-treated patients [27]. This finding is in accord with the lack of difference observed between olanzapine and risperidone in overweight inpatients in the present study.

In a randomized comparative study of aripiprazole and olanzapine, no group differences were observed in the proportions of patients with potentially clinically significant fasting HDL-cholesterol levels, because the weight gain at the study end-point was significantly greater with olanzapine than with aripiprazole [28]. The discrepancy with the present results may arise because there was no correction of HDL-cholesterol by baseline BMI, waist circumference, or weight change in their study. In a comparison between olanzapine and risperidone, the patients included across the all groups were, on average, overweight with a mean BMI of > 25 kg/m^2^ and olanzapine-treated patients had significantly lower HDL-cholesterol levels than risperidone-treated patients [29]. Because the authors could not analyze inpatients and outpatients separately, their findings may not be consistent with the results of the current study.

In a study on the acute metabolic effects of olanzapine in healthy volunteers, there was a significant decrease in HDL-cholesterol, in the absence of changes in total cholesterol and LDL-cholesterol [30]. The decrease in HDL-cholesterol within a short time period suggested a direct effect of antipsychotics on HDL-cholesterol. Birkenaes and colleagues reported that even after correction for BMI, low HDL-cholesterol was observed in patients treated with olanzapine and clozapine [31]. Similarly, Kang and colleagues showed significant differences in the prevalence of low HDL-cholesterol after adjustment for BMI among atypical antipsychotic groups [32]. There may also be differences between antipsychotics regarding the direct effects on HDL-cholesterol.

In the present study, differences in HDL-cholesterol among patients treated with antipsychotics were not detected in outpatients. Although no direct comparisons of inpatients with outpatients were made in this study, a previous study reported that hypocholesterolemia was higher in inpatients than in outpatients or the general population [33, 34]. There was a possibility that drug adherence was decreased in outpatients compared with inpatients, and thus the effects of antipsychotic drugs on HDL-cholesterol may have been decreased in outpatients. In hospitalized patients, their diet is controlled, and the proportion of obese patients is lower compared with outpatients. Therefore, in the hospitalization environment, where drug adherence and diet are controlled, it may be possible to detect differences between the effects of different antipsychotics, unlike in outpatient environments. It was also reported that the most likely improvement in the lipid profile induced by physical activity was an increase in HDL-cholesterol [35]. Because the physical activity of outpatients is likely to be higher than that of inpatients, the effects of antipsychotics on HDL-cholesterol may be masked by the influence of physical activity, which may improve HDL-cholesterol levels. Antipsychotics or physical activity may directly influence the decrease in HDL-cholesterol levels, and the differences seen between these antipsychotics may be caused by factors other than being overweight.

In the study of BMI status of hospitalized Japanese schizophrenia patients, underweight as well as obesity was a characteristic in schizophrenia inpatients compared with the general population [36]. Regarding the characteristics of underweight, a previous study showed that the prevalence of hypotriglyceridemia was significantly higher in the underweight group than in the normal weight group and overweight/obese schizophrenia inpatients [33]. In this study, we did not conduct a direct comparison of HDL-cholesterol levels between overweight, normal weight, and underweight groups. We also did not assess triglyceride levels in this study. Therefore, further studies including metabolic parameters such as triglycerides are necessary for the assessment of underweight patients with schizophrenia.

For overall patients, the olanzapine group had a statistically significant higher chlorpromazine-equivalent dosage compared with the two other groups in this study. The HDL-cholesterol levels for overweight inpatients differed significantly among the three antipsychotic groups after adjustment for chlorpromazine-equivalent dosage. Regarding the risk of metabolic syndrome in psychiatry inpatients, metabolic syndrome was associated with chlorpromazine-equivalent total daily doses, and specifically with clozapine, olanzapine, and quetiapine [37]. There was no consensus as to whether there was a dose-dependence on weight gain or metabolic effects [38].

This study reveals a difference in HDL-cholesterol in overweight Japanese inpatients with schizophrenia resulting from the use of different antipsychotics. Further studies incorporating more detailed evaluations including diet and physical activity, are needed to clarify the differences in HDL-cholesterol according to antipsychotic use.

### Limitations

The present study had several limitations. First, the cross-sectional and antipsychotic treatments were not performed randomly, and thus bias in the antipsychotic prescriptions was not controlled. Second, the present analysis was a post-hoc analysis of a study that was not designed or powered to demonstrate differences between treatments, and thus the possibility of type II errors may remain. Third, comparisons with unmedicated schizophrenia patients or healthy controls could not be performed in this study. Fourth, we did not assess psychiatric symptoms or severity using the Brief Psychiatric Rating Scale, Positive and Negative Symptom Scale or Clinical Global Impressions in this study. Psychiatric symptoms might be related to body weight, physical activity, or antipsychotic drugs, so further study including the assessment of symptoms and severity is required.

## Conclusions

This post-hoc study of a large questionnaire survey showed differences in HDL-cholesterol levels between different antipsychotic monotherapies in Japanese inpatients with schizophrenia. HDL-cholesterol was significantly lower in the olanzapine group than in the aripiprazole group in overweight inpatients. The difference was only revealed in overweight inpatients, and further study is needed to evaluate factors such as diet and physical activity to clarify the differences in HDL-cholesterol levels related to antipsychotic use.

## Abbreviations

BMI: Body mass index
DSM-IV-TR: Diagnostic and Statistical Manual of Mental Disorders, fourth edition, text revision
HDL: High-density lipoprotein
ICD-10: International Statistical Classification of Diseases and Related Health Problems version 10
LDL: Low-density lipoprotein
OECD: Organization for Economic Cooperation and Development
SD: Standard deviations
